# SUPPORT Tools for evidence-informed Policymaking in health 11: Finding and using evidence about local conditions

**DOI:** 10.1186/1478-4505-7-S1-S11

**Published:** 2009-12-16

**Authors:** Simon Lewin, Andrew D Oxman, John N Lavis, Atle Fretheim, Sebastian Garcia Marti, Susan Munabi-Babigumira

**Affiliations:** 1 Norwegian Knowledge Centre for the Health Services, P.O. Box 7004, St. Olavs plass, N-0130 Oslo, Norway; Health Systems Research Unit, Medical Research Council of South Africa; 2 Norwegian Knowledge Centre for the Health Services, P.O. Box 7004, St. Olavs plass, N-0130 Oslo, Norway; 3 Centre for Health Economics and Policy Analysis, Department of Clinical Epidemiology and Biostatistics; Department of Political Science, McMaster University, 1200 Main St. West, HSC-2D3, Hamilton, ON, Canada, L8N 3Z5; 4 Norwegian Knowledge Centre for the Health Services, P.O. Box 7004, St. Olavs plass, N-0130 Oslo, Norway; Section for International Health, Institute of General Practice and Community Medicine, Faculty of Medicine, University of Oslo, Norway; 5 Institute for Clinical Effectiveness and Health Policy, Viamonte 2146, 3rd floor, C1056ABH, Buenos Aires, Argentina; 6 Norwegian Knowledge Centre for the Health Services, P.O. Box 7004, St. Olavs plass, N-0130 Oslo, Norway

## Abstract

*This article is part of a series written for people responsible for making decisions about health policies and programmes and for those who support these decision makers*.

Evidence about local conditions is evidence that is available from the specific setting(s) in which a decision or action on a policy or programme option will be taken. Such evidence is always needed, together with other forms of evidence, in order to inform decisions about options. *Global evidence *is the best starting point for judgements about effects, factors that modify those effects, and insights into ways to approach and address problems. But *local evidence *is needed for most other judgements about what decisions and actions should be taken. In this article, we suggest five questions that can help to identify and appraise the *local evidence *that is needed to inform a decision about policy or programme options. These are: 1. What local evidence is needed to inform a decision about options? 2. How can the necessary local evidence be found? 3. How should the quality of the available local evidence be assessed? 4. Are there important variations in the availability, quality or results of local evidence? 5. How should local evidence be incorporated with other information?

## About STP

*This article is part of a series written for people responsible for making decisions about health policies and programmes and for those who support these decision makers. The series is intended to help such people ensure that their decisions are well-informed by the best available research evidence. The SUPPORT tools and the ways in which they can be used are described in more detail in the Introduction to this series *[1]. *A glossary for the entire series is attached to each article (see Additional File *1*). Links to Spanish, Portuguese, French and Chinese translations of this series can be found on the SUPPORT website http://www.support-collaboration.org. Feedback about how to improve the tools in this series is welcome and should be sent to: *STP@nokc.no.

## Scenarios

*Scenario 1: You are a senior civil servant and have responsibility for putting forward a proposal for a new health reform. You want to ensure that the proposal clearly states the number of people likely to benefit from the health reform as well as the views of stakeholder groups regarding the new initiative*.

*Scenario 2: You work in the Ministry of Health and the Minister has decided on a new health reform. You have been instructed to write a background document for the reform and need to find information on the availability of resources to implement the planned changes and possible barriers to implementation*.

*Scenario 3: You work in an independent unit that supports the Ministry of Health in its use of evidence in policymaking. You have been commissioned to write a background document for a new health reform that may affect access to care. You need to find information on access to care for the elderly and for those with low incomes in your setting*.

## Background

This article suggests a number of questions that decision makers (Scenario 1) might ask their staff to consider regarding the finding and use of evidence on local conditions to inform health policy or programme options.

The article also suggests a number of questions that those who support decision makers (Scenarios 2 and 3) should consider both when guiding the identification and appraisal of evidence from their local setting to inform a decision on health policy or programme options, and when incorporating this evidence into health policymaking.

Options should always be informed by evidence about local conditions (hereafter referred to as local evidence) together with other forms of evidence. *Global evidence *- the best evidence from around the world - is the best starting point for judgements about the effects of options and factors that modify those effects [2], and for developing insight into ways in which problems can be approached and addressed. *Local evidence *is needed for most other judgements about what decisions and actions should be taken.

Local evidence is evidence that is available from the specific setting(s) in which a decision or action on an option will be taken. The word 'local' in this instance can refer to district, regional or national levels, depending on the nature of the policy issue being considered. Such evidence might include information on the presence of factors that modify the impacts of a policy (the *modifying factors*). Such modifying factors might include: the characteristics of an area and those who live or work in it; the need for services (prevalence, baseline risk or status); views and experiences; costs; political traditions; institutional capacity; and the availability of resources such as staff, equipment and drugs.

Local evidence may be obtained from a range of sources including: routine data (e.g. on the prevalence of diseases, healthcare utilisation, or service costs); survey data (e.g. on household conditions, health and demographics); and data from one-off studies (e.g. trials conducted locally, studies of consumers' views regarding a particular health issue, and cost-effectiveness evaluations). However, local evidence is often assessed only informally or not at all as part of policymaking processes. In some settings, such information may be difficult to locate or may be of poor quality. This article provides a systematic approach to finding, assessing, and incorporating local evidence into policymaking.

There are a number of ways in which local evidence may be useful (see Table 1 for a list of some of these). For example, policymakers may need local evidence on the prevalence or magnitude of a health issue in order to contextualise (and make relevant) the evidence available from global reviews or studies conducted elsewhere [3]. (See Table 2 for a discussion of this issue in the context of malaria treatment in Tanzania and Brazil.) Evidence based on information from the global, regional or national levels may not adequately describe a local situation. Local evidence may also be useful as part of a process of priority-setting for the development of evidence-informed policy and programme options [4]. Information on local delivery, financial or governance arrangements for healthcare may be needed to inform such decisions. The views and experiences of local stakeholders, such as health professionals or consumers, regarding a particular option constitutes another important form of local evidence [5,6]. (See Table 3 for examples of how local evidence has been used in Australia for assessing needs regarding general practice, and in South Africa and Mozambique regarding views about the use of insecticide-treated nets.) Finally, information on the local costs of an option and the availability of resources is essential in taking decisions regarding implementation and in planning the delivery of options [7-9]. (See Tables 4 and 5 for examples related to this issue in South Africa, Chile and the United States.)

**Table 1:** Uses of local evidence in informing decisions on options

Local evidence can be used to:	
• Estimate the magnitude of the problem or issue that the policy aims to address	
• Diagnose the likely causes of the problem [34]	
• Contextualise, and make relevant, evidence from global reviews of the effects of interventions (e.g. by providing comparative information on the range and outcomes of interventions implemented locally)	
• Help select priorities for the development of evidence-informed policies and programmes	
• Describe local delivery, financial, or governance arrangements for healthcare	
• Inform assessments of the likely impacts of policy options (i.e. due to the existence of modifying factors)	
• Inform judgements about values and preferences regarding policy options (i.e. the relative importance that those affected attach to possible impacts of policy options) and views regarding these options	
• Estimate the costs (and savings) of policy options	
• Assess the availability of resources (including human resources, technical capacity, infrastructure, equipment) needed to implement an intervention	
• Identify barriers to implementing policy options	
• Monitor the sustainability of programme effects over time	
• Examine the effects of a policy option on particular local groups	
• Examine the equity impacts of a programme following implementation	

**Table 2:** Using local evidence to estimate the magnitude of the problem or issue that an option aims to address

A number of countries have amended their malaria policies to replace chloroquine with sulfadoxine-pyrimethamine as the first-line drug for malaria treatment, due to the growing levels of parasite resistance to chloroquine. In Tanzania, the impetus to amend treatment policies was based in part on evidence of a cure rate of approximately 40% for chloroquine, compared to 85-90% for sulfadoxine-pyrimethamine. This local evidence of the magnitude of the problem was drawn from sentinel sites across the country and linked to the growing burden of malaria morbidity and mortality observed in the country [35].	
In some Latin American countries, there is concern regarding the extent to which the pneumococcal vaccine includes the serotypes that are common in the region. In order to estimate the size of this potential problem, information from local sentinel sites has been used to evaluate the match between the serotypes included in the vaccine and those prevalent in the region. In Brazil, for example, it was estimated that 67.5% of the cases of invasive disease in children under 5 years of age were produced by serotypes included in the seven valent pneumococcal conjugate vaccine [36].	

**Table 3:** Using local evidence to inform judgements about values and views regarding options

The importance of involving consumers and communities in decisions regarding their healthcare is recognised widely. In Australia, the Consumers' Health Forum undertook consultations with consumers and consumer organisations to explore their needs and expectations regarding general practice. This evidence was gathered to inform policy development for the delivery of general practice services and the improvement of relations between key stakeholders. The evidence was fed into a number of Australian policy processes, including the government's General Practice Reform Strategy, the General Practice Strategy Review, and the development of co-ordinated care as proposed by the Council of Australian Governments [37].	
The local acceptability of community-based malaria control interventions provides another example of consumer and community involvement. Indoor residual spraying (IRS) and insecticide-treated nets - the two principal strategies for malaria prevention - are similar in cost and efficacy. The acceptability of these interventions varies across settings. In South Africa, both research and routine programme monitoring have highlighted community dissatisfaction with the IRS insecticide, DDT. This is due to the residue that DDT leaves on house walls and because it stimulates nuisance insects such as bedbugs. In certain areas of Mozambique, there are concerns that specific sleeping habits - for example, people sleeping outside due to the heat - might also negatively influence the uptake of nets [38,39].	

**Table 4:** Using local evidence to estimate the costs (and savings) of options

WHO policy recommends the use of direct observation of treatment (DOT) for treatment delivery for tuberculosis (TB). DOT can be delivered in a number of ways, including through primary healthcare clinics and in the community. An alternative policy option is for patients with TB to self-supervise their own treatment. A study was done in Cape Town, South Africa to assess the costs associated with each of the clinic, community and self-supervised options for treatment delivery. Local data were used to assess the resource input requirements of these three alternative options over a six month period of treatment. These data were then used to estimate the cost per patient treated for each of the three supervision approaches. The results indicated that the cost (in South African Rands) per patient was R3,600 for clinic supervision, R1,080 for self supervision, and R720 for community supervision. The authors concluded that community-based DOT by a volunteer lay health worker may be less costly to the health services than either clinic-based or self supervision [40]. This cost information influenced the city's decision to expand the delivery of DOT using community-based lay health workers.	
Policymakers in a Latin American country needed information on the costs of cochlear implants in order to assess the potential costs and savings of interventions to treat hearing loss. A search for local literature using Google identified a report from the Ministry of Health of Chile in which the costs were outlined for the replacement of various components needed for cochlear implants. These data were used to estimate the likely total cost of cochlear implants in the local setting. (The report can be found at: http://www.minsal.cl/ici/rehabilitacion/consentimiento_informado.pdf)	

**Table 5:** Using local evidence to assess the availability of resources with a view to informing a decision regarding options

An increasing number of countries are adding the new human papillomavirus (HPV) vaccine to routine immunisation schedules or are considering doing so. The vaccine is highly effective against the strains of the virus responsible for approximately 70% of cervical cancers and has been recommended for routine immunisation in adolescent girls in the United States. However implementation across the country is thought to be uneven. A study was undertaken in an area of North Carolina which had high rates of cervical cancer. The study explored barriers to vaccine delivery and uptake as perceived by healthcare providers. Medical practices noted a number of key concerns including: inadequate reimbursement by insurance companies of the vaccination costs, the high cost of the vaccine (given that many consumers who needed it did not have adequate health insurance), the burden on practices in ascertaining the availability of insurance cover for each patient (given the varying policies of different insurers), and the high up-front cost to practices of purchasing and storing the vaccine. The study authors note that these resource concerns may act as barriers to the implementation of the national vaccination policy [41].	

Local evidence may inform all stages of the policy process. For example, local evidence may place an issue on the policy agenda and so help to set policy goals. Local evidence may also be used by different stakeholders and interest groups to lobby for particular options. The Shack Dwellers Federation of Namibia, for example, provides support to local shack dweller associations for the collection of information on the socio-economic status of their members and other residents, and on the availability of local essential services. This information has been used to help identify local needs and also to provide local groups with a voice in government policy debates. Local groups are also able to use this information to lobby municipal officials and politicians in order to improve the quality of service provision in their areas and to make more land for housing accessible [10].

In addition to informing decisions about options directly, local evidence may be useful in monitoring the effects of a programme or policy over time in order to assess whether the anticipated impacts continue to be delivered [11]. (See Table 6 for a discussion of the use of local evidence in monitoring and evaluation in the context of antiretroviral treatment in South Africa.) Where data are collected routinely, some level of retrospective analysis may be possible and this can provide a baseline against which new programmes can be evaluated. Local evidence may also be useful in demonstrating trends in the effects of a programme across small geographic areas, such as neighbourhoods and districts, and in highlighting differences in implementation or uptake. Policymakers may also be concerned with the impacts of a programme on particular groups, such as vulnerable populations or minority groups. Local evidence may be useful in examining whether programme resources have been distributed equitably and if a programme is being implemented in ways that promote equity (see, for example, reference [12]).

**Table 6:** Using local evidence to monitor and evaluate policies

A national programme for the rollout of comprehensive HIV and AIDS care, including antiretroviral treatment (ART), has been implemented in South Africa. The Joint Civil Society Monitoring Forum - a local forum including a number of NGOs research institutes and other stakeholders - was established to assist government with the effective and efficient implementation of the programme. A briefing document outlining the lessons from this process notes that: "Democracy may be portrayed by the public's ability to contribute to and influence the state's decisions and programmes. With regard to [ART] rollout, it has been reported that access to information has been a major challenge. Reportedly not all provinces have been willing to provide information in this regard. This has made monitoring and development of appropriate resolutions difficult" ([42] p3-4). The report also highlights difficulties with obtaining disaggregated data on HIV and AIDS expenditure. It notes how these difficulties, in turn, create problems with monitoring how global HIV/AIDS budgets are being spent, particularly with regard to relative spending on treatment versus prevention, care and support [42]. This example highlights the need for local evidence to effectively monitor the implementation of a key health programme.	

Policymakers should be cautious about using local evidence alone to assess the likely impacts of policy or programme options. Local evidence may be more directly relevant than studies conducted elsewhere, but it may also be less reliable due to important limitations in the studies that were done locally. In addition, even when reliable local evaluations are available, they may be misleading because of random errors. Judgements about whether to base a conclusion on a subset of the relevant evaluations (which happen to have been undertaken locally) or on the global evidence (including relevant studies undertaken in other settings) are better informed if made in the context of a systematic review of *all *of the relevant evaluations [2].

When a systematic review is unavailable and it is not feasible to conduct or commission one, local evidence alone may be used to inform decisions about options [13]. In these circumstances, policymakers should be aware of the risks of doing this, particularly if the local evaluation has important limitations (risk of bias) or is small (and therefore the results are imprecise). However, in (the relatively uncommon) circumstances where rigorous, directly relevant and large local impact evaluations are available [14], such evidence may be optimal for informing decisions.

Like all other forms of evidence, the reliability of local evidence needs to be appraised. In this paper we suggest five questions that can help to identify and appraise local evidence that is needed to inform a decision about options.

## Questions to consider

**Figure 1:**
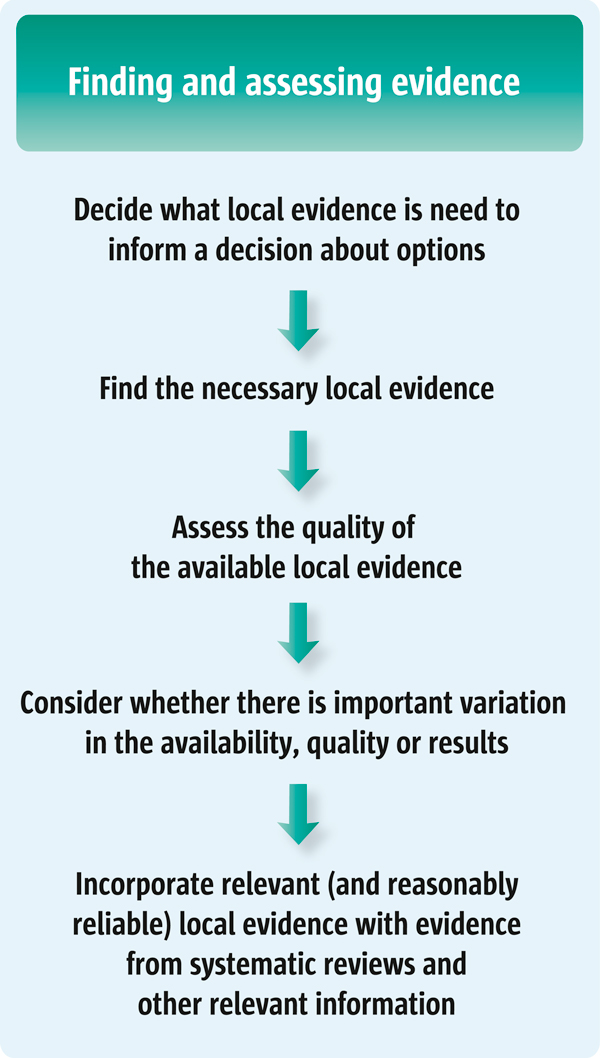
**Finding and using evidence about local conditions to inform decisions about policy or programme options**.

The following five questions can be used to guide policymakers and others in identifying potential policy and programme options and finding related evidence. The relationship between these questions is shown in Figure 1:

1. What local evidence is needed to inform a decision about options?

2. How can the necessary local evidence be found?

3. How should the quality of the available local evidence be assessed?

4. Are there important variations in the availability, quality or results of local evidence?

5. How should local evidence be incorporated with other information?

### 1. What local evidence is needed to inform a decision about options?

A range of local evidence may be needed to inform a decision about options (see Tables 1 to 8 for examples of the use of local evidence at different stages of the policy process). The evidence needed will depend on the nature of the option or question under consideration, the context, and the availability of different forms of local evidence.

**Table 7:** Using local evidence to diagnose the likely causes of a health issue

An Australian study of the factors affecting recreational physical activity found that while people living in disadvantaged areas had similar levels of access to public open space as those in wealthier locations, the equipment and space available in the disadvantaged areas were of lower quality. The study suggested that this may explain lower levels of use of these spaces in disadvantaged areas [43].	
A province in Argentina detected an increase in maternal mortality. When looking for explanatory reasons, a recent local study was identified in which the causes of maternal mortality were assessed. The report also evaluated those aspects of healthcare that needed to be modified in order to decrease mortality. This local study suggested that abortion was the most common cause of maternal death. (The report is available at: http://www.aagop.com.ar/articulos/CEDES.pdf)	

**Table 8:** Using local evidence to assess the likely impacts of options (i.e. the existence of modifying factors) and to identify barriers to implementing options

In Argentina, an evaluation was conducted of a regulation related to payments for obesity treatments, such as bariatric surgery. A national survey of cardiovascular risk factors was used to assess the extent to which obesity was a national problem. This survey provided data on the proportion of people who were overweight or obese and could therefore be used to assess the likely impacts of making different forms of obesity treatment available. (This survey is available at: http://www.msal.gov.ar/htm/Site/enfr/resultados_completos.asp)	
Canadian stakeholders participating in a deliberative dialogue about how to improve access to primary healthcare in Canada considered a variety of options. All of these included some form of transition from care which was physician-led to care which was team-led. An evidence brief, drawing on local evidence, was prepared to inform the dialogue. This identified four potential barriers to the implementation of the options:	
1. Initial wariness among some patients of potential disruptions to their relationship with their primary healthcare physician	
2. Wariness on the part of physicians of potential infringements on their professional and commercial autonomy, in the light of the private delivery component of the 'private delivery/public payment' arrangement with physicians	
3. A potential lack of viability in terms of organisational scale in many rural and remote communities, and	
4. Government willingness to extend public payment to other healthcare providers and teams while at the same time maintaining the existing public payment to physicians, as part of the 'private delivery/public payment' arrangement with physicians. This was considered to be a particular concern during a recession [44]	

### 2. How can the necessary local evidence be found?

Local evidence may be obtained from routine health information systems, from larger surveys or studies that can be disaggregated, or from specific studies that have collected or analysed data on a local level. We discuss each of these in more detail below.

Like those processes related to global evidence of effects [15], the processes of searching for local evidence and making judgements regarding its inclusion and assessment should be systematic (i.e. systematic processes should be used to ensure that relevant research is identified, appraised and used appropriately) and should also be reported transparently. The selective use of local evidence (sometimes referred to as 'cherry picking') to demonstrate the usefulness of a particular option, should be avoided as it may result in important data or information being omitted or overlooked during the decision making process. For example, including only the largest estimates of the size of a problem, such as the proportion of children who do not complete their vaccination schedule, will result in a poor understanding of a problem such as incomplete vaccination. It may also result in scarce resources being allocated to interventions that are not needed, that do not respond to local needs, or that may not be needed at the extent to which they are provided. Using the largest estimates of the proportion of children who do not complete their vaccination schedule to inform a decision regarding options, for example, may result in more resources being allocated to the vaccination programme than are actually needed. Similarly, relying only on data on average immunisation coverage across a large population to inform policy may be inadequate. Such evidence may conceal large inequities in coverage across specific areas or groups.

While a wide range of sources of local evidence may be available, this evidence may not be available in a form that addresses the policy question under consideration. For example, data may be available from a survey on household access to different forms of sanitation, such as flush toilets or pit latrines. However, these data may not have been analysed at the level of aggregation needed, such as a specific health district or region, and may not indicate whether the sanitation facilities were operational. It may therefore be necessary to undertake further analysis of available data or to make assumptions regarding the applicability of the data to a particular policy question. We discuss this further in Questions 4 and 5 below.

#### Local collected data obtained from the routine health information system

National, district, or other local health authorities (or other organisations in the health system) often collect data routinely on a wide range of issues, including [16]:

• *Risk factors*: Such as nutrition and blood pressure

• *Mortality and burden of disease*: This includes health outcomes such as child mortality, TB treatment outcomes, peri-operative deaths, infectious disease and cancer notifications

• *Health service coverage*:

∘ Coverage for clinical interventions or services such as childhood vaccinations or cervical screening rates

∘ Health service utilisation information such as length of hospital stay, number of outpatient visits for specific health conditions, and prescription drugs dispensed

∘ Routine surveys of patient satisfaction with care

• *Health systems resources*:

∘ Healthcare expenditures according to various cost centres and programmes

∘ Human resource data such as numbers and grades of staff in different facilities and programmes, staff development programmes delivered, and staff absenteeism

∘ Clinical performance data such as post-surgical infection rates, time to treatment for people with myocardial infarctions

∘ Guidelines used for care delivery

∘ Adherence to guidelines for care delivery

• *Inequities in healthcare and health outcomes*

For some of these sources, it may be possible to disaggregate data by specific groups, such as gender or age, or by specific local area, such as a neighbourhood or town [2]. Data from routine health information systems may not have been analysed systematically and considerable resources may be needed to undertake such analysis.

Good starting points for identifying local sources of routine data include the Health Information Departments of Ministries of Health, National Statistics Offices, and local health authorities. Increasingly, these departments publish lists of the range of data that they capture and analyse on the Internet. Many also regularly produce summary statistics. The City of Cape Town Health Department in South Africa, for example, publishes information on their website by sub-district for a small range of health indicators, such as number of live births, number of infant deaths, infant mortality rates, TB case loads and treatment outcomes (see: http://www.capetown.gov.za/en/cityhealth/Pages/CityHealth.aspx). The Association of Public Health Observatories also provides data on key health indicators for each local authority in England (see: http://www.apho.org.uk/default.aspx?QN=P_HEALTH_PROFILES). Local research institutions, health non-governmental organisations (NGOs), or the offices of bilateral or multi-lateral agencies, such as WHO country offices, may also be able to advise on local sources of routinely collected data. Some commercial databases may include useful local evidence, for example, related to local prices for drugs, their availability, and the use of other technologies. In general, local health authorities should maintain an overview of local sources of routinely collected data. Policymakers may want to familiarise themselves with these.

#### Data from larger surveys or studies that can be disaggregated to local level

Important data sources include large surveys or studies such as national censuses, regional surveys of access to basic facilities, and national demographic and health surveys. For some of these sources, disaggregation to the provincial or city level may be possible or may already have been conducted. For example, the Neighbourhood Statistics site of the United Kingdom Office for National Statistics (see: http://www.neighbourhood.statistics.gov.uk/dissemination/ allows users to find statistics for an area by entering its name or postcode. Data on a wide range of topics are available, including access to services, crime and safety, general health, and teenage pregnancies. Similarly, the website of Statistics South Africa includes information on a wide range of topics disaggregated to a provincial level. For example, this includes information, based on data from a national household survey, on health insurance coverage and health service consultations by province (see: http://www.statssa.gov.za).

For other datasets, analysis to the appropriate local level may not be conducted routinely. This may be feasible, though, if data are tagged by geographic area. The agency that conducted the survey or the agency housing these data should be able to advise on whether further disaggregation to the local level is possible. The process of further analysis is more complex and statistical support is therefore generally recommended. Some health data, such as the use of treatment services for sexually transmitted infections and HIV/AIDS, may be considered sensitive in nature. It may therefore not be possible to obtain data disaggregated to a local level if the agencies housing these data need to ensure that specific individuals cannot be identified from information placed in the public domain.

#### Specific studies that have collected and analysed data on a local area

Large numbers of research studies collect, analyse and report data focused on a local area such as a province of a country or a city. These studies may use a wide range of data collection and analysis methods. Studies that present data on a local area can be located in several ways:

• By searching (ideally with the help of an information specialist) global databases of published research papers, such as PubMed, the Cochrane Library or the WHO regional databases (e.g. the Latin American and Caribbean Health Sciences Database [LILACS]), using geographic terms such as 'Caracas' or 'Buenos Aires'. PubMed includes a *hedge*, or validated search strategy, that allows users to search for administrative databases studies, community surveys and qualitative studies (these may be helpful in providing information on utilisation patterns and on views and experiences, for example). This is available at: http://www.nlm.nih.gov/nichsr/hedges/search.html

• By searching (ideally with the help of an information specialist) sources of 'grey' or unpublished literature, such as Google Scholar, the WHO Library Information System http://dosei.who.int/uhtbin/cgisirsi/Mon+May++4+21:00:46+MEST+2009/0/49, and OpenSIGLE (System for information on grey literature in Europe: http://opensigle.inist.fr). Many local studies, such as operational research on health services, are published as reports on the web but may not be published in research journals. Grey literature is therefore a good source of such evidence

• By contacting local researchers in universities, research institutes or health departments or local research networks for relevant information, including unpublished study reports

• By contacting or searching the resources of health observatories such as the European Observatory on Health Care Systems http://www.euro.who.int/observatory, the International Observatory on Mental Health Systems http://www.cimh.unimelb.edu.au/iomhs, or the Africa Health Workforce Observatory http://www.afro.who.int/hrh-observatory

### 3. How should the quality of the available local evidence be assessed?

Like all other forms of evidence, the *quality *of local evidence needs to be assessed. Where data quality is poor, interpretation can be difficult and there is a danger that faulty conclusions may be drawn. When considering local evidence, it may be useful to differentiate between *data *(i.e. the raw product of measurements or observations) and *information *(i.e. data that are organised or analysed in relation to a specific question or issue and are therefore more useful for decision making [17]). Some of the potential problems with local evidence relate to data (e.g. the ways in which measurement was done). Others relate to *how *these data are converted into information (e.g. as part of the analysis process).

A number of factors may compromise the quality of routinely-collected local data. Healthcare workers who collate and enter data, for example, may be poorly trained in this task. Similarly, if they do not receive timely feedback, they may not understand the usefulness of the data to informing service delivery. Data entry may also compete with a large number of other care tasks in clinics or hospitals and central quality control may be inadequate [18]. Problems related to the quality of data may be difficult to rectify once data have been collected. In contrast, it may be easier to rectify inadequacies in information by re-running an analysis. Systems for the collection of local data should ideally be designed to provide useful and timely feedback of information to those who collect such data.

Most local evidence that is used to inform decisions about options is *descriptive *(i.e. it includes simple summaries of the sample and measures or outcomes included in the data) rather than *comparative *(i.e. based on the comparison of one set of data with another, for example by area or over time). There are some exceptions, such as evidence about inequities which relies on comparisons.

The descriptive nature of most local evidence has implications for assessing its quality. In the case of comparative studies, the assessment of quality is focused primarily on the risk of bias (i.e. the risk of "a systematic error, or deviation from the truth, in results or inferences" [19]). In contrast, key questions in assessing the quality of local evidence include the following (adapted from [11]. Also see Table 9 for a summary of questions that can be used to guide assessments of the quality of local evidence):

**Table 9:** Questions to guide assessment of the quality of local evidence

Main quality criteria	Sub-questions	Example of the assessment of the quality of local evidence: routinely collected data on TB treatment outcomes from TB Registers
Is the evidence representative?	• Is there a clear description of the source of the evidence? • If the evidence is drawn from a sample of the population of interest, is there a clear description of how the sampling was conducted? • Was the sampling approach appropriate (where applicable)? • Is there a description of how any inferences or generalisations were made to the wider population?	TB Registers should routinely record information on each patient diagnosed with TB. The information is not based on a sample of the population of interest. It should therefore be representative of the demographics and treatment outcomes for people with TB in a particular setting, provided that it is completed for each person with TB

Is the evidence accurate?	• Is there a clear description of who collected the data? • Were the data collectors appropriately trained and supported in this task? • What tools were used for data collection? • Were appropriate tools used? • When were the data collected? • Was the quality of the data collected monitored and was the quality shown to be adequate? • How were the data analysed? • Was the method of analysis reported clearly? • Were any data limitations discussed?	Most health authorities provide a manual, based on WHO guidance, for completion of the TB Register. This generally specifies what information should be collected and by whom. In using these data, policymakers need to check whether there is clear guidance on completion of the Register, whether TB programme staff have been trained in its use, whether there are mechanisms in place to check the quality of the data at clinic and district levels, and whether data compilation was done appropriately

Are appropriate outcomes reported?	• Is there a clear description of the outcome/s measured? • Is the outcome measure reliable? • Were these outcomes measured appropriately? • Do these outcomes provide a reasonable assessment of the health issue?	A standard range of measures is generally included in TB Registers, based on WHO guidance. These are designed to assess the functioning of the TB programme. However, the data do not generally provide direct measures of issues such as patient satisfaction with the care provided by TB programme staff

• *Is the evidence representative? *This question focuses on whether the evidence correctly represents the wider population from which it is drawn or to which the findings are generalised. There are several components to this question: firstly, is there a clear description of the source of the evidence? Secondly, if the evidence is drawn from a sample of the population of interest, is there a clear description of how the sampling was conducted, and was the sampling approach that was used appropriate? Thirdly, is there a description of how any inferences or generalisations were made to the wider population?

• *Is the evidence accurate? *This question is concerned with whether the available data match, or are likely to match, the actual value of the outcome measured. When addressing this question, the user may want to consider whether there are clear descriptions of the processes through which the data were collected. Issues that should be addressed include: who collected the data and were they appropriately trained and supported in this task, what tools were used for data collection, when were the data were collected, was the quality of the collected data monitored, how was the analysis done (were the methods of analysis reported clearly), and were any data limitations discussed

• *Are appropriate outcomes reported? *This question focuses on whether the measures reported in the data (such as treatment outcomes or health utilisation measures) are suitable for addressing the question for which the data will be used. When addressing this question, the user may want to consider whether there is a clear description of the outcome or outcomes measured, whether they are reliable, and whether these outcomes will provide a reasonable assessment of the health issue. If policymakers are considering, for example, how to improve the quality of care for people with TB, routinely-reported TB treatment outcomes may be a useful measure. This is because the completion of TB treatment is likely to be related to the quality of care received by patients

### 4. Are there important variations in the availability, quality or results of local evidence?

When assessing and using local evidence, it is important to be aware of variations in its availability, quality or results. Each of these issues is discussed below.

#### Availability

Large variations always occur in the range or depth of available local evidence across geographic areas, jurisdictions or population groups. In many instances, this variation may simply reflect differences in the policies or capacity of health authorities or other agencies across different jurisdictions or areas. In some cases, however, variations in the availability of local evidence across groups or areas may reflect other underlying inequities. These may include the poor access that certain groups have to health facilities, or the failure of surveys to include 'hard to reach' groups such as migrant populations, those speaking other languages, or those living in remote or poorly serviced areas. Groups that are stigmatised on the basis of ethnicity or sexual orientation, for example, or because they are viewed as illegal migrants, may also be reluctant to identify themselves as belonging to these groups for the purposes of data collection [20,21]. There may therefore be little available local evidence related to these groups and collecting such data may be very challenging. Those using local data need to explore the reasons for variations in its availability and consider such factors in the decision making process.

Availability may be limited in other ways. Firstly, evidence may be available from only one source, making it difficult to cross-check the information's reliability. Secondly, information may be available for a large area that includes the area of policy interest but in a form that does not allow this local area information to be separated from the wider dataset. Thirdly, policymakers may have access to good quality data from a neighbouring area and may then have to assess the extent to which these data can be generalised to the area of interest. Finally, local evidence may be available only for an indicator assessing a related health issue. For example, policymakers in Colombia required data on the number of hospitalisations for meningitis but this information was not available routinely. However, the number of deaths due to meningitis in Columbia was available from the WHOSIS information system http://apps.who.int/whosis/database/mort/table1.cfm. In addition, data on meningitis mortality rates were available from a local source http://www.scielo.br/pdf/rsap/v8s1/v8s1a04.pdf. From these two sets of data, it is possible to estimate the total number of meningitis cases in the country.

#### Quality and results

Different sources of local evidence may differ in quality. In addition, the quality of local evidence may differ from that of other forms of evidence used in decision making. For example, a study of routine malaria data in Mozambique compared paper-based district records of adult inpatient malaria cases and deaths with digital data captured at the provincial level. Large discrepancies between these sources of data were identified (a 62% difference for cases and a 48% difference for deaths). The authors suggested that these variations may be related to errors in the data entry process at the provincial level [22]. Such differences in data quality should be considered explicitly in the decision making process.

Variations in the results of local evidence on a particular health issue across sources of local evidence may occur for a number of reasons, including:

• Differences in the way in which the issue was defined and measured across the sources

• Differences between the individuals, groups or other entities about whom data were collected across the sources

• Differences in the comparators used

• Differences (where applicable) in the interventions delivered

• Differences in the ways in which data were collected and analysed across the sources

When considering such variations, users of these data should explore the following questions:

• Is the variation potentially important from a clinical or policy perspective?

• If the variation is important, is a reasonable explanation clear from the data sources, or can a reasonable explanation be hypothesised (e.g. differences in recruitment, measurement, analysis etc.)?

• Are there other sources of information against which the local evidence can be compared?

Users of data should document any decisions they take regarding the interpretation of the evidence and should note any uncertainties, as discussed below.

### 5. How should local evidence be incorporated with other information?

Policy decisions require a combination of *global evidence *(the best available evidence from around the world) - ideally from systematic reviews - and different types of *local evidence*, assumptions and judgements. When local evidence is key to a policy decision (i.e. it might influence a decision in one direction or another) it is important to:

• Describe the approach used to *identify *the local evidence. Ideally a systematic approach to accessing this evidence should be used

• Describe the approach used to *assess *the local evidence. As noted earlier, a systematic approach to assessing evidence is recommended. When shortcuts are necessary, or it is necessary to make assumptions or use informal observations, these should be made transparent

• Describe clearly what local evidence is used and from where the evidence is obtained. This should include detail related to the specific groups or communities from which the evidence is drawn. As far as possible, documents and other sources should be cited and made available to others involved in the decision making process

• Describe any important gaps or uncertainties in the evidence due to the lack of local information or its poor quality. A study of the use of data available from the national Australian Childhood Immunization Register, for example, found that there were challenges in using the Register to adequately measure immunisation rates and outcomes in specific populations, such as remote indigenous groups [13]. Similar uncertainties have been reported from LMICs [23,24]. There may also be uncertainties in evidence due to conflicting findings between different sets of local evidence. For example, hospital mortality rates, complication rates, or duration of stay in intensive care may all be used to assess the quality of surgical care. Studies have found a poor correlation between these different indicators [18,25,26]. Consequently, it may be difficult to decide which set of data best reflects the 'real' quality of surgical services in a hospital or region and therefore which dataset should be used to inform policymaking. The applicability of local evidence to particular population subgroups may also be uncertain. For example, local evidence on teenage pregnancy rates may be available for the general population but not available by population subgroups (e.g. by ethnicity or language)

• Finally, it is important to identify and discuss any differences between the findings obtained from *global *evidence and those obtained from *local *evidence. For example, global evidence suggests that lay health workers can be effective in improving the uptake of immunisation in children [27]. However, local evidence might suggest otherwise if there are strong local views that lay people are inadequately qualified to provide health advice. In this instance, the promotion of this cadre would be less effective locally. Such local evidence might lead to less confidence (i.e. greater uncertainty) about the applicability of global evidence on lay health workers for immunisation uptake, even though the global review would still be seen as providing the best available estimate of effectiveness. Caution also needs to be used in applying economic evidence from other settings to a particular jurisdiction as the relative costs of some inputs may vary greatly across settings. For example, human resource costs generally vary locally while pharmaceutical costs may be similar across settings.

A good understanding of the local context and conditions may be helpful in interpreting both local and global evidence [28]. Key elements of context that should be considered include: the physical context (such as health facilities, supply chains, banking systems, etc.), human resources, knowledge (including the skills to implement a policy or intervention), the socio-cultural context (including issues such as belief systems, values, corruption, etc.), and the political context. Tools such as political mapping may be useful in developing an understanding of political context [29,30].

Approaches such as rapid appraisal can be used to bring together the range of different data available at the local and global levels to address a specific policy question. For example, this approach has been used to draw together data related to the management of diabetes care in Georgia and in Kyrgyzstan [31,32]. Local evidence, together with an appraisal of its reliability, may also be incorporated into policy briefs and a range of other documents that are used to inform policy processes. We discuss the use of policy briefs in more detail elsewhere [33].

## Conclusion

Local evidence may inform all stages of the policy process - from influencing the policy agenda through to shaping programme choices and monitoring programme sustainability (see Table 10 for examples of the types of local evidence that might be relevant to specific policy questions). Such evidence may be obtained from routine health information systems, from surveys or studies that can be disaggregated, or from studies in which data have been collected or analysed on a local level. Both the evidence needed and the evidence available will depend on the nature of the policy question under consideration and the context.

**Table 10:** Types of local evidence to address specific policy questions

Stage of the policy cycle	Use of local evidence	Types of local evidence that might be relevant
Diagnosing the problem or goal	To estimate the magnitude of the problem or issue that the policy aims to address and stakeholders' views on it	• Vital statistics data from routine sources, surveys such as the national DHS • Morbidity data from routine sources at national, sub-national or institutional (e.g. hospital) level • Local studies of stakeholder views and experiences
	
	To diagnose the likely causes of the problem	• Local studies of stakeholder views and experiences • Data on risk factors from surveys
	
	To describe local delivery, financial or governance arrangements for healthcare	• Ministry of Health and Ministry of Finance policies, guidelines and records • Regulations of professional organisations

Assessing policy options	To contextualise evidence from global reviews of the effects of interventions and to make this evidence relevant	• Data from local health delivery agencies on the range of interventions currently implemented (for a particular health problem) and their outcomes, which can be compared with the programmes evaluated in global reviews • Data from local health delivery agencies on local coverage of these interventions
	
	To inform assessments of the likely impacts of policy options (e.g. due to the existence of modifying factors)	• Local studies of similar programmes
	
	To inform judgements about values and preferences regarding policy options (i.e. the relative importance that those affected attach to possible impacts of policy options) and views regarding these options	• Local studies of stakeholder views • Information from stakeholder organisations, e.g. organisations representing the public and specific consumer groups, such as those living with particular health problems • Information from deliberative dialogues with stakeholders
	
	To estimate the costs (and savings) of the policy options	• Local studies of programme costs and savings • Cost data held by health departments or programmes or by non-governmental delivery agencies
	
	Examine the effects of a policy option on particular local groups	• Routinely collected programme data • Local studies focusing on the group/s of interest

Exploring implementation strategies for a policy option	To assess the availability of resources (including human resources, technical capacity, infrastructure, and equipment)	• Resource data held by health departments or programmes or by non-governmental delivery agencies • Local studies of resource use by similar programmes
	
	To identify barriers to implementing policy options	• Local studies of stakeholder views • Information from stakeholder organisations, e.g. organisations representing the public and specific consumer groups, such as those living with particular health problems • Information from deliberative dialogues with stakeholders • Local barrier studies

Monitoring the effects of a policy option	Monitor the sustainability of programme effects over time	• Routinely collected programme data
	
	Examine the equity impacts of a programme following implementation	• Data that can be disaggregated by gender, age, area of residence, etc.

In many settings, steps need to be taken to improve the quality and use of data about local conditions. These may include motivating data collectors by ensuring that such information is useful to them and fed back in a timely way. It may also be necessary to ensure that policymakers and those who support them are aware of the sources of data about local conditions. As with other forms of evidence, the quality of local evidence needs to be assessed. Policymakers should be cautious about using local evidence alone to assess the likely impacts of policy or programme options. Local evidence may be more directly relevant than studies conducted elsewhere. But it may also be less reliable due to the important limitations of studies that are undertaken locally.

## Resources

### Useful documents and further reading

- WHO. World Health Statistics. Indicator compendium (Interim version). Geneva: World Health Organisation. 2009 http://www.who.int/whosis/indicators/en/.

- The 'Creating Excellence' network in the United Kingdom has produced a short local evidence guide and a toolkit on gathering and analysing local level data. http://www.creatingexcellence.org.uk/regeneration-renewal-news262.html

- Department for Education and Skills, United Kingdom. Using local evidence. A leaflet for service managers, planners and commissioners. http://www.dcsf.gov.uk/everychildmatters/_download/?id=5728

### Links to websites

WHO Statistical Information System (WHOSIS): http://www.who.int/whosis/en - This is an interactive database bringing together core health statistics for the 193 WHO Member States. It comprises more than 100 indicators, which can be accessed by way of a quick search, by major categories, or through user-defined tables.

African Index Medicus: http://indexmedicus.afro.who.int - An international index to African health literature and information sources produced by the WHO in collaboration with the Association for Health Information and Libraries in Africa. It provides access to health information published in, or related to, Africa and can be searched at no cost.

The Cochrane Library: http://www3.interscience.wiley.com/cgi-bin/mrwhome/106568753/HOME - The Cochrane Library contains high-quality, independent evidence to inform healthcare decision making. It includes reliable evidence from Cochrane and other systematic reviews and clinical trials. Cochrane reviews provide the combined results of the world's best medical research studies and are recognised as the gold standard in evidence-based healthcare.

PubMed: http://www.ncbi.nlm.nih.gov/pubmed - The PubMed database contains more than 19 million citations for biomedical articles from a wide range of indexed journals and can be searched at no cost.

Health Metrics Network: http://www.who.int/healthmetrics/en - A global partnership on health information system strengthening. The website provides a range of tools and information to support health information system strengthening.

Demographic and health survey data: http://www.measuredhs.com - The demographic and health surveys programme has collected, analysed and disseminated data on population, health, HIV and nutrition through more than 200 surveys in over 75 countries. The website provides a range of freely available data from these surveys.

## Competing interests

The authors declare that they have no competing interests.

## Authors' contributions

SL prepared the first draft of this article. ADO, JNL, AF, SGM and SMB contributed to drafting and revising it.

## Supplementary Material

Additional file 1 Glossary Click here for file
